# Editorial: Imaging biomarkers in neurodegenerative diseases: advances and challenges

**DOI:** 10.3389/fnagi.2026.2009935

**Published:** 2026-10-09

**Authors:** Francesco Lanfranchi, Andrew R. Bender, Lucas Caldi Gomes

**Affiliations:** 1 Department of Health Sciences (DISSAL), University of Genoa, Genoa, Italy; 2 Cleveland Clinic Lou Ruvo Center for Brain Health, Las Vegas, NV, United States; 3 NanoTemper Technologies GmbH, Munich, Germany

**Keywords:** cognitive impairment, functional near-infrared spectroscopy, magnetic resonance imaging, movement disorders, neurodegenerative diseases, optical coherence tomography, positron emission tomography

Neurodegenerative diseases comprise biologically and clinically heterogeneous disorders whose underlying changes evolve over time and cannot be fully characterized by any single biomarker. Imaging offers complementary ways to investigate these processes *in vivo*, but its value ultimately depends on methodological robustness, biological interpretability, and validation in representative populations. The contributions included in this Research Topic, “*Imaging biomarkers in neurodegenerative diseases: advances and challenges*,” reflect the breadth of current research in neurodegenerative imaging, spanning different diseases, biological targets, and methodological approaches. Figure 1 provides an overview of the imaging modalities and neurodegenerative conditions explored in this Research Topic.

**Figure 1:**
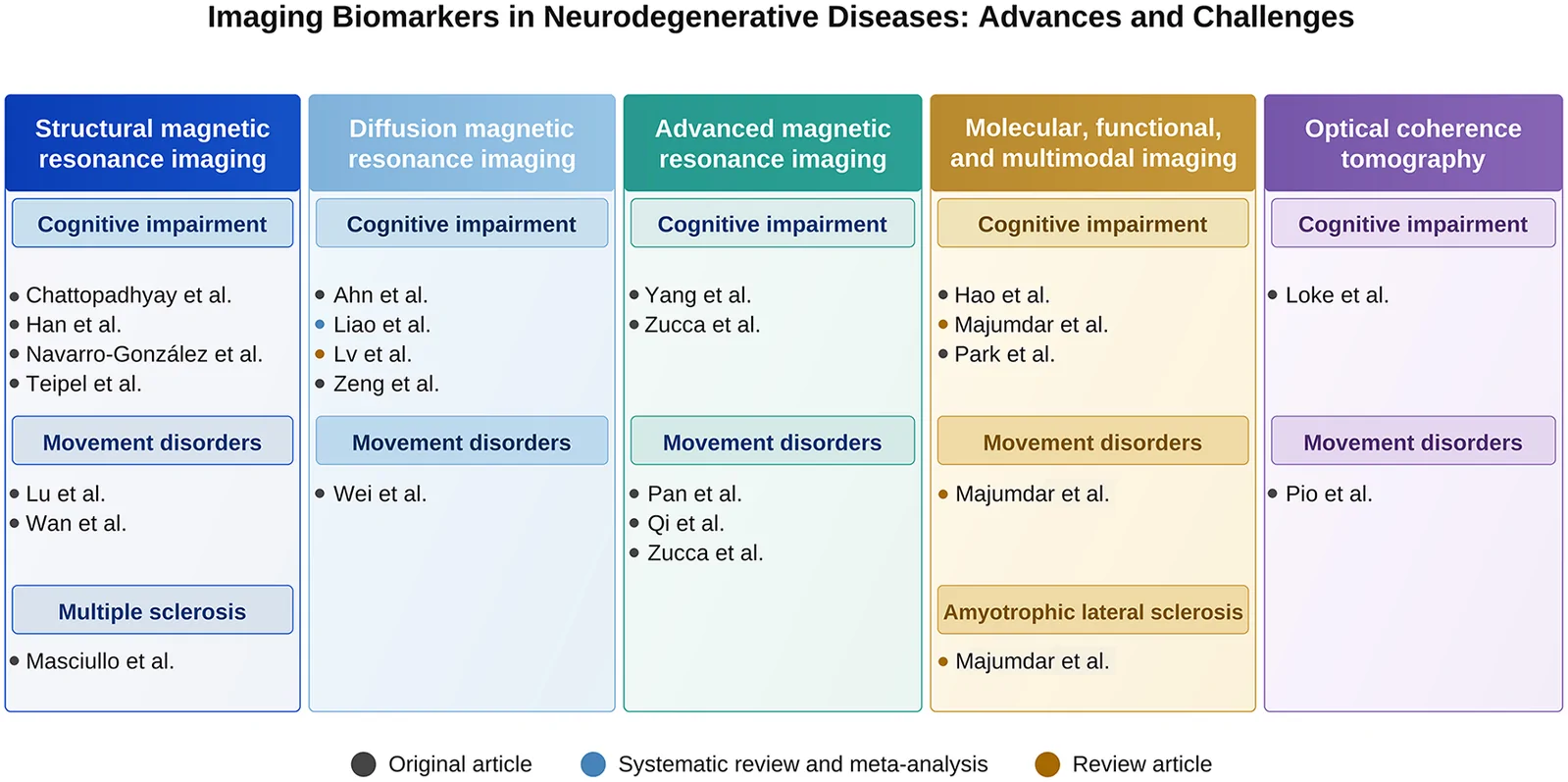
Overview of the imaging modalities and neurodegenerative conditions explored in the Research Topic.

Structural magnetic resonance imaging (MRI) was the most widely represented approach, reflecting both its accessibility and its central role in quantitative neurodegeneration research. Navarro-González et al. first addressed a practical methodological question by comparing ultra-fast and conventional three-dimensional (3D) T1-weighted MRI acquisitions in a real-world cognitive disorders clinic including Alzheimer's disease and frontotemporal lobar degeneration. Brain-age estimates were broadly consistent across protocols, although the degree of interchangeability varied across analytical pipelines, indicating that shorter acquisitions may be feasible without assuming that all processing methods respond identically. Moving from acquisition efficiency to clinically relevant associations, Teipel et al. examined whether anticholinergic medication burden was related to cognition and longitudinal atrophy across the Alzheimer's disease spectrum. The association was clearer for baseline global cognition than for subsequent decline or structural change.

A second group of studies used regional and subregional morphometry to improve anatomical specificity. Han et al. combined olfactory testing with hippocampal and amygdala subregional morphometry derived from 3D T1-weighted MRI across healthy controls, mild cognitive impairment, and Alzheimer's disease. Differences in selected hippocampal subfields were detectable even when total hippocampal volume was not, while olfactory performance worsened across diagnostic groups. In Parkinson's disease, Wan et al. found that entorhinal cortex atrophy measured on structural MRI statistically mediated the associations of plasma phosphorylated tau (p-tau) 181, glial fibrillary acidic protein (GFAP), and neurofilament light chain (NfL) with cognition, with some relationships differing according to cognitive status. Lu et al. extended this structural MRI subregional approach to the amygdala, identifying selective involvement of medial and cortical nuclei in Parkinson's disease with mild cognitive impairment, whereas no significant amygdala subregional atrophy was detected in subjective cognitive decline. Considered together, these studies show how anatomically refined measurements can reveal patterns that may be obscured in whole-structure analyses.

Structural imaging was also applied to less frequently studied disorders and to the development of more robust quantitative tools. Lopez-de-Ipina et al. used 3-T structural MRI with voxel- and surface-based morphometry and found no case-control differences in essential tremor that survived correction for multiple comparisons, despite subtler uncorrected findings in cerebellar and motor-related regions, thereby illustrating the difficulty of identifying stable structural signatures in a clinically heterogeneous disorder. In multiple sclerosis, Masciullo et al. evaluated an automated measure of upper cervical spinal cord cross-sectional area derived from MRI that used a fixed pontomedullary junction reference, avoiding dependence on vertebral labeling, and remained associated with neurological disability. Finally, Chattopadhyay et al. used data from three large cohorts to compare deep-learning and automated machine-learning models integrating 3D T1-weighted MRI with clinical and demographic data for predicting future cognitive decline. Their results showed that MRI did not consistently improve prediction when clinical and demographic variables already provided a strong baseline, indicating that multimodal model complexity did not necessarily translate into greater predictive performance.

Diffusion MRI shifted the emphasis from macroscopic anatomy to tissue microstructure and perivascular water transport. In Parkinson's disease, Wei et al. used diffusion MRI with free-water imaging to examine the nucleus basalis of Meynert in relation to olfactory function. Free water remained associated with worse olfactory performance after adjustment for age, whereas between-group differences in free water and volume were not statistically significant. The findings therefore support an association between diffusion-derived tissue properties and olfactory dysfunction in the absence of significant between-group differences in nucleus volume. At a broader disease level, Lv et al. reviewed concurrent gray- and white-matter changes across the Alzheimer's disease continuum, emphasizing that white-matter volume loss and microstructural disruption contribute alongside cortical and subcortical atrophy.

Three contributions focused on imaging markers related to perivascular spaces. Zeng et al. compared diffusion tensor imaging analysis along the perivascular space (DTI-ALPS) with a diffusion kurtosis imaging-based ALPS (DKI-ALPS) approach. Both measures were reduced in cognitive impairment, with the kurtosis-based index showing a larger group difference and stronger associations with longitudinal trajectories, amyloid burden, and cognitive measures. Ahn et al. compared DTI-ALPS with the conductivity tensor imaging-based CTI-ALPS index, but observed limited separation across normal cognition, amnestic mild cognitive impairment, and Alzheimer's disease, while conventional diffusion-based measures showed stronger clinical associations. Liao et al. then placed these findings in a broader context through a systematic review and meta-analysis, identifying progressively lower DTI-ALPS index values across normal cognition, mild cognitive impairment, and Alzheimer's disease, although marked between-study heterogeneity constrained direct clinical interpretation and the certainty of the evidence was rated as low.

Advanced MRI expanded the range of biological processes assessed beyond conventional morphometry and diffusion. Pan et al. combined hippocampal morphometry derived from structural MRI with resting-state functional MRI (fMRI) connectivity in *de novo* Parkinson's disease across normal cognition, subjective cognitive decline, and mild cognitive impairment. The differing structural and functional patterns across cognitive groups suggest that the two measurements provide complementary information. Qi et al. similarly combined modalities by integrating arterial spin labeling and quantitative susceptibility mapping with machine learning. Perfusion and susceptibility measures separated Parkinson's disease from healthy controls more clearly than they distinguished patients with and without cognitive impairment. The combined machine-learning models achieved high classification performance.

Other studies addressed the biological meaning and clinical implementation of advanced MRI contrasts. Zucca et al. directly investigated the tissue basis of neuromelanin-sensitive MRI in post-mortem midbrain specimens from cases of Parkinson's disease and Alzheimer's disease, showing that both neuromelanin and iron contributed independently to the measured signal. This provides important biological validation while also demonstrating that a single imaging contrast may reflect multiple tissue components. Yang et al., by contrast, focused on automated image interpretation and developed a deep-learning system for detecting cerebral microbleeds on routinely acquired two-dimensional T2^*^-weighted gradient-echo MRI. The study responds to the growing need for efficient assessment of hemorrhagic lesions relevant to amyloid-related imaging abnormalities, while positioning automated detection as a support tool rather than a substitute for expert review.

The contributions based on molecular, functional, and multimodal imaging explored how complementary sources of information can be integrated. Hao et al. combined cerebellar radiomic features from structural MRI and [^1^8F]fluorodeoxyglucose positron emission tomography (PET), and the multimodal model outperformed either single-modality model in distinguishing Alzheimer's disease from cognitively normal participants. This result supports the potential of cross-modality fusion. Park et al. instead used functional near-infrared spectroscopy (fNIRS) during cognitive training in older adults with amnestic mild cognitive impairment. Greater medial prefrontal activation during attentional training was associated with more favorable subsequent cognitive change, suggesting that task-related functional measurements may help characterize individual variability in training responsiveness rather than diagnosis alone. Given the small sample and exploratory design, however, the finding is best regarded as hypothesis-generating.

The two review articles in this section broadened the discussion beyond individual applications. Mao et al. provided a narrative overview of imaging strategies relevant to the proposed bone–brain axis in cognitive decline and Alzheimer's disease, linking structural and functional brain imaging, molecular imaging, and quantitative assessment of bone density and microarchitecture. Majumdar et al. offered a broader narrative synthesis of structural MRI, diffusion imaging, functional MRI, PET, hybrid PET/MRI, and emerging molecular targets across Alzheimer's disease, Parkinson's disease, frontotemporal dementia, and amyotrophic lateral sclerosis. Although their scopes differed, both reviews converged on the value of combining modalities that capture distinct aspects of disease biology.

Optical coherence tomography (OCT) introduced a retinal perspective to the Research Topic and highlighted the appeal of low-burden, widely accessible imaging. Loke et al. examined acquisition artifacts in a longitudinal memory-clinic cohort and found that their presence was associated with subsequent cognitive decline and dementia. The observation raises the possibility that irregularities usually treated only as technical noise may also reflect visuomotor, attentional, or behavioral instability during image acquisition, supporting their further evaluation as an opportunistic prognostic signal. In Parkinson's disease, Pio et al. used spectral-domain OCT and identified selective thinning of the temporal-inferior sector of the peripapillary retinal nerve fiber layer compared with healthy controls, while differences between motor phenotypes were not robust after correction for multiple comparisons and associations with cognition, motor severity, and dopaminergic treatment remained exploratory. Together, these studies illustrate the potential of OCT for investigating retinal and acquisition-related features associated with neurodegeneration.

Taken together, the contributions in this Research Topic illustrate the expanding role of imaging in neurodegenerative diseases and its potential to capture clinically and biologically relevant aspects of disease. At the same time, several recurring challenges remain, including methodological heterogeneity, the need for greater standardization of acquisition and analytical approaches, and the importance of longitudinal, multicenter, and external validation. Addressing these issues will be essential to improve the reproducibility, generalizability, and clinical applicability of emerging imaging biomarkers and to clarify their added value alongside established clinical and imaging measures.

